# Differential Effects of Endotracheal Suctioning on Gas Exchanges in Patients with Acute Respiratory Failure under Pressure-Controlled and Volume-Controlled Ventilation

**DOI:** 10.1155/2015/941081

**Published:** 2015-03-31

**Authors:** Xiao-Wei Liu, Yan Jin, Tao Ma, Bo Qu, Zhi Liu

**Affiliations:** ^1^Department of Emergency, The First Affiliated Hospital of China Medical University, Shenyang 110001, China; ^2^Department of Biostatistics, School of Public Health, China Medical University, Shenyang 110001, China

## Abstract

This study was conducted to evaluate the effects of open endotracheal suctioning on gas exchange and respiratory mechanics in ARF patients under the modes of PCV or VCV. Ninety-six ARF patients were treated with open endotracheal suctioning and their variations in respiratory mechanics and gas exchange after the suctions were compared. Under PCV mode, compared with the initial level of tidal volume (*V*_*T*_), ARF patients showed 30.0% and 27.8% decrease at 1 min and 10 min, respectively. Furthermore, the initial respiratory system compliance (*C*_rs_) decreased by 29.6% and 28.5% at 1 min and 10 min, respectively. Under VCV mode, compared with the initial level, 38.6% and 37.5% increase in peak airway pressure (PAP) were found at 1 min and 10 min, respectively. Under PCV mode, the initial PaO_2_ increased by 6.4% and 10.2 % at 3 min and 10 min, respectively, while 18.9% and 30.6% increase of the initial PaO_2_ were observed under VCV mode. Summarily, endotracheal suctioning may impair gas exchange and decrease lung compliance in ARF patients receiving mechanical ventilation under both PCV and VCV modes, but endotracheal suctioning effects on gas exchange were more severe and longer-lasting under PCV mode than VCV.

## 1. Introduction

Respiratory failure is an acute or chronic condition with impaired gas exchange and pulmonary functions and is characterized by elevated carbon dioxide or decreased oxygen in the arterial blood [1]. Respiratory failure can result from diverse conditions such as cardiac and respiratory diseases, defects in neuromuscular systems that control breathing, injury to chest, and several lung diseases. Importantly, respiratory failure is associated with a high morbidity and mortality [2, 3]. The majority of patients with respiratory failure exhibit shortness of breath, and the low oxygen and high carbon dioxide levels in the blood can impair heart and brain functions [4, 5]. Currently, treatment for respiratory failure includes clearing the airways by suction, use of bronchodilators, or tracheostomy and endotracheal tube with ventilator support. Additionally, the treatment regimen includes antibiotics for infections, anticoagulants for pulmonary thromboembolism, and electrolyte replacement for fluid imbalance [4]. Mechanical ventilation is a method to mechanically assist or replace spontaneous breathing to cure respiratory failure [6]. Mechanical ventilation is effective in improving gas exchange, while reducing dyspnea and inspiratory effort in patients with respiratory failure, and averts risks secondary to endotracheal intubation [7, 8].

Due to establishment of artificial airway, most acute respiratory failure (ARF) patients receiving mechanical ventilation could not produce sputum. Suctioning could effectively eliminate airway secretions to maintain respiratory function [9]. Endotracheal suctioning is required to avert accumulation of secretions and its related complications and to maintain airway patency [10]. During open endotracheal suctioning, gas aspirated from the lung will facilitate the movement of secretions towards the suction catheter [11]. However, ARF patients are separated from the ventilator during open endotracheal suctioning, which may decrease positive airway pressure (PAP) and lung volume [4]. Alveolar collapse may induce a further decline in lung volume during negative-pressure suction [12], thereby affecting gas exchange and respiratory mechanics in ARF patients [2]. This study investigated the effects of open endotracheal suctioning on respiratory mechanics and gas exchange in ARF patients under PCV and VCV.

## 2. Methods

### 2.1. Ethics Statement

The Ethics Committee of the First Affiliated Hospital of China Medical University approved this study. Written informed consent was acquired from all subjects using the procedures authorized by institutional review boards. Next of kin, caretakers, or guardians consented representing the study participants whose capacity to consent was reduced. The study was executed at the emergency intensive care units (EICU) at the First Affiliated Hospital of China Medical University.

### 2.2. Study Design and Subjects

This was a single center prospective study of acute respiratory failure (ARF) patients admitted to the EICU between October 2010 and February 2013. Patients met the following criteria to be enrolled in this study; patients lived up to the criteria for the diagnosis of ARF: (1) type I: PaO_2_ < 60 mmHg, PaCO_2_ < 50 mmHg; (2) type II: PaO_2_ < 60 mmHg, PaCO_2_ ≥ 50 mmHg. All patients received endotracheal intubation and continuous mechanical ventilation (Maquet, Servo V.2.0, Germany), continuous monitoring of heart rate (HR), blood pressure (BP), and SpO_2_ with Philips Intellivue MP60 monitor (Philips, Medizinsysteme, Germany).

In the present study, ninety-six acute respiratory failure (ARF) patients (45 males and 51 females) were enrolled, with a mean age of 62 years (range: 26~84 years). They were divided into two groups: PCV and VCV. PCV group included 49 patients (25 males and 24 females), with a mean age of (57 ± 21) years. And they were with an APACHE II score of (17.6 ± 10.3), a disease course of (4.6 ± 2.0) years. There were 15 patients who had complications, such as compensatory chronic respiratory failure, acidosis, pulmonary encephalopathy, and gastrointestinal bleeding. Compared with PCV, VCV group had 47 patients (20 males and 27 females), with a mean age of (58 ± 26) years. Their APACHE II score was (17.2 ± 10.1) and disease course was (5.1 ± 2.2) years. Twenty among them had complications, including compensatory chronic respiratory failure, acidosis, pulmonary encephalopathy, and gastrointestinal bleeding. As shown in Table 1, there was no statistical difference in gender ratio, age, APACHE II score, disease course, and complications between the two groups of subjects (all *P* > 0.05).

### 2.3. Protocol

The 96 ARF patients were randomly divided into the PCV and VCV groups using a random number generator. Diazepam (10 mg) was injected intravenously before suctioning. A standardized method was used for a standardized lung volume method after 1 h of mechanical ventilation: (1) sufficient suctioning for mouth, nasal, and airway secretions; (2) 10 cm H_2_O (0.98 kPa) plateau airway pressure (*P*_plat_) by increasing tidal volume (*V*_*T*_) under VCV mode and 10 cm H_2_O inspiratory pressure under PCV mode, maintaining ventilation and oxygenation for 20 s. According to the clinical need for suctioning, suction indications include visible secretions attached within the patient's endotracheal tube, auscultation of lung breath weak sound and sputum sound, patients with irritability and other poor performances, changes, or abnormities in breathing rate, slowness of heart rate, increase in blood pressure, reduction of ventilation, and detection of SpO_2_ less than 90%. Endotracheal suctioning was performed when alarm for peak airway pressure (*P*_peak_) under VCV mode, low tidal volume under PCV, or SpO_2_ decreased more than 5%. During endotracheal suctioning, endotracheal tube departed from Y-shaped tube. The 12F suction catheter (4 mm exterior diameter) was inserted into endotracheal tube about 30 cm after blocking negative pressure suction and then slowly rotated and the suction catheter was withdrawn at 150 mmHg. Endotracheal tube was connected with ventilator tube for mechanical ventilation after suctioning.

### 2.4. Outcome Measurements

The HR, mean arterial pressure (MAP), *V*_*T*_, *P*_peak_, and *P*_plat_ were recorded at 15 min after standardized lung volume (baseline), before suctioning, and 1, 3, 5, and 10 min after suctioning. Total respiratory system compliance (*C*_rs_) was reckoned by [*C*_rs_ = *V*_*T*_/(*P*_peak_ − PEEP(5–8 cm H_2_O))]. Values of *V*_*T*_, *P*_peak_, and *P*_plat_ were the average of five respiratory cycles. Dynamic changes in SpO_2_ were monitored. Arterial blood gas analysis was performed at baseline, before suctioning, and at 3 and 10 min after suctioning.

### 2.5. Statistical Analysis

Data was presented as mean ± standard deviation (SD), median with interquartile ranges (IQR), or frequencies. A *χ* ^2^ test was used to compare frequencies. One-way analysis of variance (ANOVA) and Student's* t*-test were applied to generally distributed variables, while the Mann-Whitney *U* test was applied to nonnormal distributed variables. Comparisons between two groups for nominal variables were made by the Fisher exact test. All of the statistical significance tests were two-sided, with a *P* value less than 0.05 considered as statistically significant. All the statistical analysis was performed by the use of SPSS 18.0 software (SPSS, Inc., Chicago, IL, USA).

## 3. Results

### 3.1. Changes in Hemodynamic Parameters

Changes in heart rate (HR) and mean arterial pressure (MAP) before and after suctioning at different time points under PCV and VCV modes are shown in Table 2. Under PCV mode, the HR and MAP showed an increase of 10.5% and 11%, respectively, at 5 min after suctioning, compared with the baseline level (HR: 105 ± 17 versus 95 ± 13 minute^−1^, *P* < 0.05; MAP: 91 ± 14 versus 82 ± 12 mmHg, *P* < 0.05, resp.). Under VCV mode, the HR and MAP increased by 10.4% and 11.1%, respectively, at 5 min after suctioning compared with the baseline level (HR: 106 ± 21 versus 96 ± 17 minute^−1^, *P* < 0.05; MAP: 90 ± 12 versus 81 ± 10 mmHg, *P* < 0.05, resp.). No significant differences were found in changes in HR and MAP at 10 min after suctioning compared with the baseline level under both PCV and VCV modes (all *P* > 0.05). Furthermore, there were also no significant differences in HR and MAP between PCV and VCV modes at different time points after suctioning (all *P* > 0.05).

### 3.2. Changes in Respiratory Mechanics

**Figure 1:**
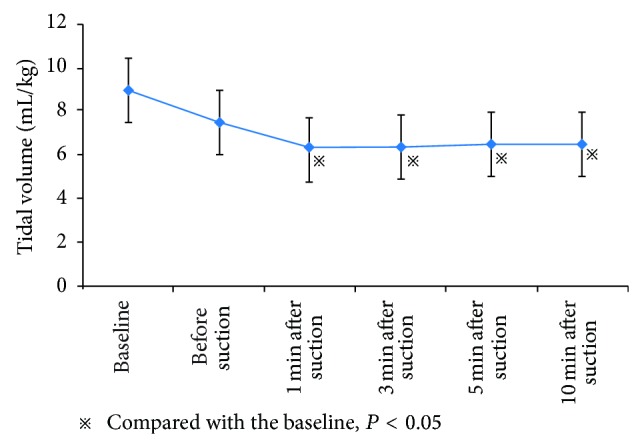
Changes in tidal volume (*V*_*T*_) under PCV mode of ARF patients before and after suctioning at different time points.

**Figure 2:**
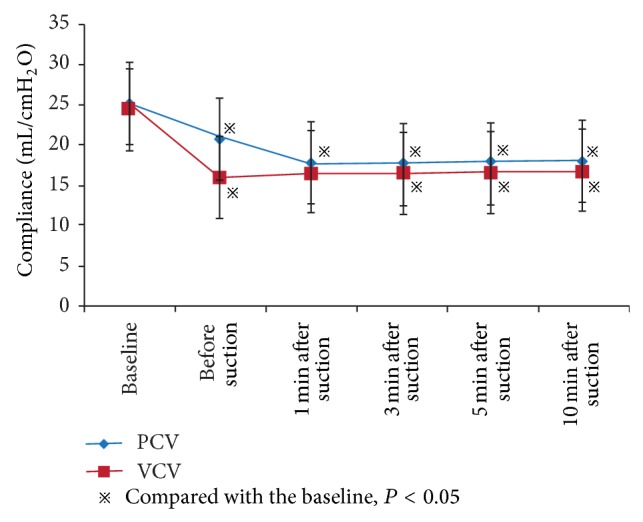
Changes in respiratory system compliance (*C*_rs_) of ARF patients before and after suctioning at different time points under PCV and VCV modes.

Changes in tidal volume (*V*_*T*_), *C*_rs_, and airway pressure in ARF patients before and after suctioning at different time points are shown in Table 3. Under PCV mode, the *V*_*T*_ and *C*_rs_ showed a decrease of 30.0% and 29.6%, respectively, at 1 min after suctioning, compared with the baseline level (*V*_*T*_: 6.3 ± 1.8 versus 9.0 ± 0.1 mL/kg, *P* < 0.05; *C*_rs_: 17.8 ± 6.8 versus 25.3 ± 7.8 mL/cm H_2_O, *P* < 0.05, resp.) (Figures 1-2). At 10 min after suction, the *V*_*T*_ and *C*_rs_ decreased by 27.8% and 28.5%, respectively (*V*_*T*_: 6.5 ± 2.0 versus 9.0 ± 0.1 mL/kg, *P* < 0.05; *C*_rs_: 18.1 ± 7.3 versus 25.3 ± 7.8 mL/cm H_2_O, *P* < 0.05, resp.). No significant differences were found for changes in the *V*_*T*_ and *C*_rs_ at 10 min after suctioning, compared with baseline level (all *P* > 0.05).

**Figure 3:**
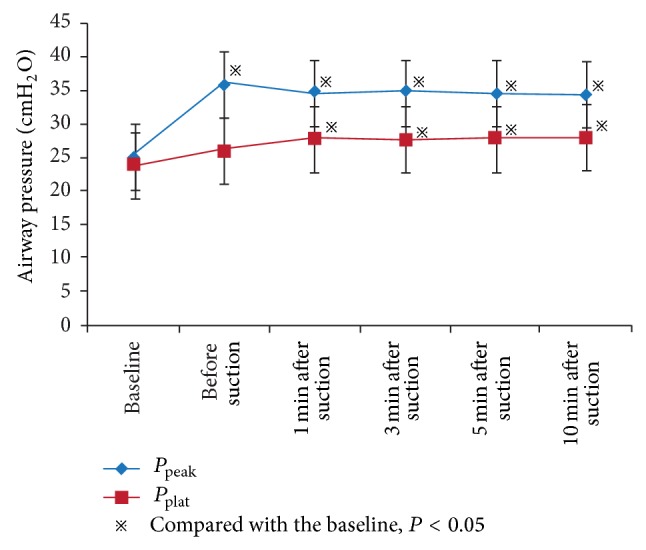
Changes in airway pressure of ARF patients before and after suctioning at different time points under VCV mode.

Under VCV mode, peak airway pressure (*P*_peak_) and plateau pressure (*P*_plat_) showed an increase of 38.6% and 17.2%, respectively, at 1 min after suctioning, compared with the baseline level (*P*_peak_: 34.8 ± 8.4 versus 25.1 ± 7.5 cm H_2_O, *P* < 0.05; *P*_plat_: 27.9 ± 7.3 versus 23.8 ± 5.8 cm H_2_O, *P* < 0.05, resp.), while *C*_rs_ decreased by 32.5% (16.6 ± 5.1 versus 24.6 ± 6.3 mL/cm H_2_O, *P* < 0.05) (Figures 2-3). At 10 min after suctioning, the *P*_peak_ and *P*_plat_ showed an increase of 37.5% and 17.6%, respectively (*P*_peak_: 34.5 ± 8.2 versus 25.1 ± 7.5 cm H_2_O, *P* < 0.05; *P*_plat_: 28.0 ± 7.6 versus 23.8 ± 5.8 cm H_2_O, *P* < 0.05, resp.), while *C*_rs_ decreased by 31.7% (16.8 ± 5.5 versus 24.6 ± 6.3 mL/cm H_2_O, *P* < 0.05). Compared with the level before suction, *t* changes in the *P*_peak_ and *C*_rs_ at 10 min after suctioning also did not represent notable differences (all *P* > 0.05).

### 3.3. Changes in Gas Exchange

**Figure 4:**
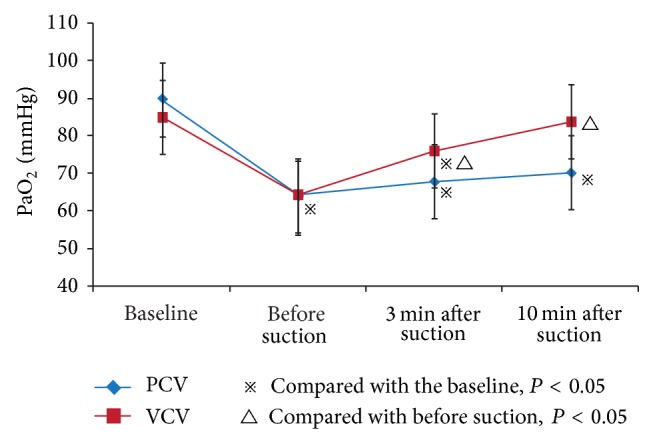
Changes in PaO_2_ of ARF patients before and after suctioning at different time points under PCV and VCV modes.

Under PCV mode, compared with the initial level before suctioning, PaO_2_ showed an increase of 6.4% and 10.2% at 3 and 10 min, respectively, after suctioning (3 min: 67.7 ± 13.2 mmHg versus 63.6 ± 14.1 mmHg, *P* > 0.05; 10 min: 70.1 ± 18.7 mmHg versus 63.6 ± 14.1 mmHg, *P* > 0.05, resp.) (Figure 4). However, there was still a prominent difference in PaO_2_ between the baseline level and the level at 10 min after suctioning (70.1 ± 18.7 mmHg versus 89.6 ± 15.1 mmHg, *P* < 0.05). Under VCV mode, compared with the level before suction, PaO_2_ also showed an increase of 18.9% and 30.6% at 3 and 10 min, respectively, after suctioning (3 min: 76.2 ± 13.6 mmHg versus 64.1 ± 13.2 mmHg, *P* < 0.05; 10 min: 83.7 ± 16.9 mmHg versus 64.1 ± 13.2 mmHg, *P* < 0.05, resp.). There was no obvious difference in PaO_2_ between the baseline level and the level at 10 min after suctioning (83.7 ± 16.9 mmHg versus 85.1 ± 14.2 mmHg, *P* > 0.05). We have demonstrated that under PCV mode PaO_2_ had a mean increase (3 min: (6.4 ± 2.6) %; 10 min: (10.2 ± 6.2) %) for ARF patients after suctioning compared with before suctioning, and the increased ratio was lower than under VCV mode (all *P* < 0.05).

## 4. Discussion

Mechanical ventilation mechanically assists or replaces spontaneous breathing [13]. In general, mechanical ventilation should be considered when there are clinical or laboratory signs that the patient cannot maintain an open airway or adequate oxygenation or ventilation [14]. Endotracheal suctioning is a frequently performed procedure for acute respiratory failure patients receiving mechanical ventilation [15]. Endotracheal suctioning can avoid accumulation of secretions, tracheal occlusion, increased work of breathing, atelectasis, and pulmonary infections, thereby ensuring optimal oxygenation or ventilation [16]. However, endotracheal suctioning may also cause opposite effects, such as arrhythmia, hypoxemia, airway and environment microbial contamination, and ventilator-associated pneumonia (VAP) [16]. Furthermore, endotracheal suctioning may lead to different effects on gas exchange and respiratory mechanics in acute respiratory failure patients under the mode of volume-controlled ventilation (VCV) and pressure-controlled ventilation (PCV). Open suctioning requires the ventilator to be disconnected and many patients experience anxiety and fear during suction, which results in resentment and irritability and potential psychological stress disorders. The closed circuit suctioning maintains the connection with the ventilator, giving patients the required sense of security. By the comparison of the open suctioning and closed circuit suctioning methods, these evidences supported that closed circuit suctioning is superior to open suctioning [17]. Under VCV mode, increase of airway pressure was in response to reduced compliance, increased resistance, or active exhalation and may increase ventilator-induced lung injury risk. PCV inhibits most of airway pressure delivered to the lung but may lead to changeable tidal and minute volume [18]. However, there are few studies on differential effects of endotracheal suctioning in acute respiratory failure patients under PCV and VCV.

In the present study, we evaluated endotracheal suctioning effects on gas exchange and respiratory mechanics in 96 ARF patients under PCV and VCV modes. Our results indicated that endotracheal suctioning under PCV and VCV modes impairs both gas exchange and respiratory mechanics in ARF patients receiving mechanical ventilation. Compared with the initial level of *V*_*T*_, ARF patients showed a 30.0% and 27.8% decrease at 1 min and 10 min, respectively, after suctions under PCV mode. Alveolar collapse under PCV mode could lead to an increased respiratory resistance when the inspiratory pressure was stabilized on a fixed point, thereby causing *V*_*T*_ to reduce [19]. Furthermore, the initial *C*_rs_ decreased by 29.6% and 28.5% at 1 min and 10 min, respectively, after suctions under PCV mode. The major causes of suctioning effects on respiratory mechanics include alveolar collapse, lung volume, and compliance decrease [20]. Under VCV mode, a 38.6% increase and 37.5% increase of PAP were found, compared with the initial level, at 1 min and 10 min, respectively, after suctions. We also found a 17.2% and 17.6% increase of *P*_plat_ and a 32.5% and 31.7% decrease of *C*_rs_ at 1 min and 10 min, respectively, after suctions under VCV mode. Given that *V*_*T*_ under VCV mode was not changed, alveolar collapse would lead airway pressure to increase accordingly and overexpansion of the open alveolar may also cause increase in *P*_plat_.

During mechanical ventilation, the retention of airway mucus causes increased airway resistance, which is manifested as increased airway pressure [21]. However, we found no significant airway pressure decrease after suctioning under VCV mode. The possible explanation could be that, in general, the insertion depth of the suction tube could only explore carina and bronchus to effectively remove the mucus of the proximal airway, while the leading factors that influence airway resistance are the small bronchi rather than the carina and bronchus. Due to the bronchial spasms resulting from stimulation of suction tube, along with other factors such as alveolar collapse and atelectasis, airway resistance after suction was not lower than the original level, which was presented as an increased *P*_peak_ under VCV mode and a decreased *V*_*T*_ under PCV mode. In addition, our results also suggested that the initial PaO_2_ increased by 6.4% and 10.2% at 3 min and 10 min, respectively, after suctions under PCV mode, while 18.9% increase and 30.6% increase of the initial PaO_2_ were observed under VCV mode. There was a significant difference in the increment of the initial PaO_2_ between PCV and VCV modes. Our results are consistent with multiple previous studies [22–25].

In conclusion, our study provides a comprehensive and reliable evidence that endotracheal suctioning of ARF patients receiving mechanical ventilation may impair gas exchange and decrease lung compliance, under both PCV and VCV modes, but the effects of endotracheal suctioning on gas exchange were more severe and longer-lasting under PCV mode, in comparison to VCV mode.

## Figures and Tables

**Table 1:** Comparison of patients' general information between the two groups.

General information	PCV (49)	VCV (47)	*t*/*F*	*P*

Gender (M/F)	25/24	20/27	0.379	0.538
Age	57 ± 21.1	58 ± 26.2	0.207	0.836
APACHE II	17.6 ± 10.3	17.2 ± 10.1	0.192	0.848
Disease course	4.6 ± 2.0	5.1 ± 2.2	1.165	0.247
Complications	15	20	1.124	0.289

M: male; F: female; APACHE: acute physiology and chronic health evaluation.

**Table 2:** Changes in heart rate (HR) and mean arterial pressure (MAP) before and after suctioning at different time points under PCV and VCV modes.

Time	PCV		VCV	
	HR (beat/min)	MAP (mmHg)	HR (beat/min)	MAP (mmHg)

Basic level	95 ± 13	82 ± 12	96 ± 17	81 ± 10
Before suctioning	117 ± 16	86 ± 13	112 ± 15	86 ± 14
1 min	122 ± 17^*^	89 ± 15^*^	109 ± 12^*^	88 ± 16^*^
3 min	108 ± 15^*^	90 ± 17^*^	104 ± 12^*^	89 ± 12^*^
5 min	105 ± 17^*^	91 ± 14^*^	106 ± 21^*^	90 ± 12^*^
10 min	100 ± 14	87 ± 13	98 ± 17	84 ± 14

^*^Compared with the baseline, *P* < 0.05; HR: heart rate; MAP: mean arterial pressure; PCV: pressure-controlled ventilation; VCV: volume-controlled ventilation.

**Table 3:** Tidal volume (*V*_*T*_), respiratory system compliance (*C*_rs_), and airway pressure changes before and after endotracheal suctioning under pressure-controlled ventilation (PCV) or volume-controlled ventilation (VCV).

Time	PCV		VCV		
	*V*_*T*_ (mL/kg)	*C*_rs_ (mL/cmH_2_O)	*P*_peak_ (cmH_2_O)	*C*_rs_ (mL/cmH_2_O)	*P*_plat_ (cmH_2_O)

Baseline level	9.0 ± 0.1	25.3 ± 7.8	25.1 ± 7.5	24.6 ± 6.3	23.8 ± 5.8
Before suctioning	7.5 ± 2.2^*^	20.9 ± 5.8^*^	36.2 ± 8.7^*^	15.9 ± 5.8^*^	26.2 ± 6.4
1 min	6.3 ± 1.8^*^	17.8 ± 6.8^*^	34.8 ± 8.4^*^	16.6 ± 5.1^*^	27.9 ± 7.3^*^
3 min	6.4 ± 1.9^*^	17.8 ± 6.3^*^	34.9 ± 6.5^*^	16.6 ± 6.6^*^	27.8 ± 5.9^*^
5 min	6.5 ± 1.6^*^	18.0 ± 6.7^*^	34.7 ± 7.8^*^	16.7 ± 6.4^*^	27.9 ± 6.9^*^
10 min	6.5 ± 2.0^*^	18.1 ± 7.3^*^	34.5 ± 8.2^*^	16.8 ± 5.5^*^	28.0 ± 7.6^*^

^*^ *P* < 0.05, compared with the baseline level, PCV: pressure-controlled ventilation, VCV: volume-controlled ventilation, *V*_*T*_: tidal volume, *P*_peak_: airway peak pressure, *C*_rs_: respiratory system compliance, and *P*_plat_: airway plat pressure.
